# Ecological specialization and rarity indices estimated for a large number of plant species in France

**DOI:** 10.1016/j.dib.2015.02.015

**Published:** 2015-03-04

**Authors:** Samira Mobaied, Nathalie Machon, Emmanuelle Porcher

**Affiliations:** Centre d׳Ecologie et des Sciences de la Conservation (CESCO, UMR7204), Sorbonne Universités, MNHN, CNRS, UPMC, CP51, 55 rue Buffon, 75005 Paris, France

**Keywords:** Ecological specialization indices, Rarity, Species co-occurrence, Flora of France

## Abstract

The biological diversity of the Earth is being rapidly depleted due to the direct and indirect consequences of human activities. Specialist or rare species are generally thought to be more extinction prone than generalist or common species. Testing this assumption however requires that the rarity and ecological specialization of the species are quantified. Many indices have been developed to classify species as generalists vs. specialists or as rare vs. common, but large data sets are needed to calculate these indices.

Here, we present a list of specialization and rarity values for more than 2800 plant species of continental France, which were computed from the large botanical and ecological dataset SOPHY. Three specialization indices were calculated using species co-occurrence data. All three indices are based on (dis)similarity among plant communities containing a focal species, quantified either as beta diversity in an additive (Fridley et al., 2007 [6]) or multiplicative (Zeleny, 2008 [15]) partitioning of diversity or as the multiple site similarity of Baselga et al. (2007) [1]. Species rarity was calculated as the inverse of a species occurrence.

**Specifications table**

**Table:** 

Subject area	Conservation ecology
More specific subject area	Ecological specialization and rarity indices
Type of data	Table
How data was acquired	Specialization and rarity indices were computed from the large botanical and ecological database SOPHY (GIVD ID EU-FR-003) using R 3.0.1 [13] and the code provided by Fridley et al. [6] in Appendix S1, Zeleny [15] in Appendix S2 and Baselga et al. [1] via the *R* function Simpson.multi (×). Rarity was calculated as the inverse of species occurrence.
Data format	Analyzed
Experimental factors	N/A
Experimental features	N/A
Data source location	France
Data accessibility	Data are provided in the paper

## Value of the data

• This paper provides a list of specialization and rarity indices for over 2800 plants species in France.
• The ecological specialization indices are based on species co-occurrence and are considered as important indicators quantifying both species response to the environment and interactions among species.
• The data provided in the paper open new perspectives to study species response to global changes [3], for example to examine the process of floristic homogenization [11,12].

## 1 Materials and methods

### 1.1 Data sets

To calculate specialization and rarity indices we used the botanical and ecological database SOPHY (France GIVD ID EU-FR-003 [7]). This database compiles over 200,000 surveys of vascular plant species recorded in France between 1915 and 2010 in a wide variety of habitats (mostly in natural or semi-natural habitats such as forests, meadows and grasslands, but also in anthropogenic habitats such as crop fields [7]). The nomenclature used in the SOPHY database and in this paper follows Bock [2]. We merged all vegetation layers and worked with presence–absence data for all species. Specialization and rarity indices were calculated only for species observed in at least 50 plots (2879 species out of a total of 5267 species in the database; 135,002 plots).

### 1.2 Specialization Index

To calculate species ecological specialization we chose a class of indices based on species co-occurrence, with the following assumption: generalist species, which can grow in a wide variety of environmental conditions, should co-occur with many species, while specialist species, which tolerate only a narrow range of environmental conditions, should co-occur with relatively fewer species (see e.g. [6]). As highlighted by Devictor et al. [4], such metric quantifies the response of species to a range of environmental variables, and thereby describes the breadth of the Grinnellian niche [9], but also incorporates interactions among species, thereby characterizing the Eltonian niche breadth as well [5]. In this data paper, we provide three different indices of species specialization based on species co-occurrence; the latter is quantified via the beta-diversity of plant communities containing a given species. Fridley et al.׳s [6] index uses additive partitioning (see below). Zeleny [15] however observed that additive partitioning caused species specialization to depend on the size of the species pool at the position of species optima. To correct this, he recommended replacing additive with multiplicative diversity partitioning. Manthey and Fridley [10] finally emphasized the usefulness of the multiple Simpson approach in the estimation of species niche width. The three indices were thus calculated as follows.

The index of Fridley et al. [6], *θ*_*b*_, is

$$\theta_{b=\gamma -\mu \left(\alpha \right)}$$

where *γ* is the cumulative number of species over all plots and *µ*(*α*) is mean plot species richness. Following Fridley et al. [6], *θ*_*b*_ was calculated 100 times for each species, each time on a random subset of 50 plots in which the species was observed. In these 50 plots, we retained all species observed in the surveys, including rare species observed in fewer than 50 plots and for which no specialization index was calculated. We provide the mean *θ*_*b*_ for each species in supplementary Table 1.

The second species specialization index, *θ*_*wb*_, was obtained using Whittaker׳s beta [14], i.e. a multiplicative partitioning of diversity (supplementary Table 2):

$$\theta_{wb=\gamma /\mu \left(\alpha \right)}$$

Finally, following Manthey and Fridley [10] we calculated for each species the multiple-site similarity measure from the Simpson index, using the method of Baselga et al. [1] (supplementary Table 3):

$$M_{sim=}\frac{\sum_{i}S_{i}-S_{T}}{\left[\sum_{i<j}\;\min\left(b_{ij},b_{ji}\right)\right]+\left[\sum_{i}S_{i}-S_{T}\right]}$$

where *S*_*i*_ is the total number of species in site *i*, *S*_*T*_ is the total number of species in all sites, and *b*_*ij*_ and *b*_*ji*_ are the number of species that occur in site *i* (respectively *j*), but not site *j* (respectively *i*).

Species rarity was calculated as the inverse of occurrence, following Gaston [8], i.e.

*Rar*=1/(number of plots where a plant species is present). The values are normalized between 0 and 1 (data in supplementary Table 4).

These three indices of ecological specialization, which use the same general approach, are of course significantly correlated with each other. Note that large values of $\theta_{b}$ and $\theta_{wb}$, two measures of dissimilarity among plant communities, are expected to indicate more generalist species, whereas the reverse is true for $M_{sim}$. The latter specialization index is therefore negatively correlated with $\theta_{b}$ and $\theta_{wb}$ (Table 1). Finally, there is a general tendency for specialist species to be also rare species, especially for the $M_{sim}$ specialization index (negative correlation between rarity and $\theta_{b}$ or $\theta_{wb}$ and strong positive correlation between rarity and $M_{sim}$, Table 1).

## Figures and Tables

**Table 1:** Correlations among specialization and rarity indices. The table contains Pearson׳s correlation coefficients below diagonal and *R*² values above. All correlation coefficients are significantly different from 0 (*P*<0.0001).

	$\theta_{b}$	$\theta_{\mathrm{wb}}$	$M_{\mathrm{sim}}$	**Rarity**
$\theta_{b}$	–	*R*²=0.12	*R*²=0.17	*R*²=0.11
$\theta_{\mathrm{wb}}$	0.44	–	*R*²=0.24	*R*²=0.12
$M_{\mathrm{sim}}$	−0.41	−0.49	–	*R*²=0.76
**Rarity**	−0.33	−0.44	0.87	–

## Supplementary materials

Table 1. Index of species ecological specialization using additive partitioning of diversity ($\theta_{b}$) for 2879 plants species in France.Table 2. Index of species ecological specialization using multiplicative partitioning of diversity ($\theta_{wb}$) for 2879 plants species in France.Table 3. Index of species ecological specialization using multiple-site similarity measure from the Simpson index ($M_{sim}$) for 2879 plants species in France.Table 4. Species rarity for 2879 plants species in France.
